# Development of immunohistochemistry and in situ hybridisation for the detection of SARS-CoV and SARS-CoV-2 in formalin-fixed paraffin-embedded specimens

**DOI:** 10.1038/s41598-020-78949-0

**Published:** 2020-12-14

**Authors:** Fabian Z. X. Lean, Mart M. Lamers, Samuel P. Smith, Rebecca Shipley, Debby Schipper, Nigel Temperton, Bart L. Haagmans, Ashley C. Banyard, Kevin R. Bewley, Miles W. Carroll, Sharon M. Brookes, Ian Brown, Alejandro Nuñez

**Affiliations:** 1 Pathology Department, Animal and Plant Health Agency (APHA), Woodham Lane, New Haw, Addlestone, KT15 3NB UK; 2 Department of Viroscience, Erasmus Medical Centre, Rotterdam, The Netherlands; 3 Virology Department, Animal and Plant Health Agency (APHA), Woodham Lane, New Haw, Addlestone, KT15 3NB UK; 4 Viral Pseudotype Unit, Medway School of Pharmacy, Universities of Greenwich and Kent at Medway, Chatham, ME4 4TB UK; 5 National Infection Service, Public Health England, Porton Down, Salisbury, SP4 0JG UK; 6 School of Life Sciences, University of Sussex, Falmer, Brighton, BN1 9QG UK; 7 Institute for Infection and Immunity, St George’s Hospital Medical School, University of London, London, SW17 0RE UK

**Keywords:** SARS-CoV-2, Microscopy

## Abstract

The rapid emergence of SARS-CoV-2, the causative agent of COVID-19, and its dissemination globally has caused an unprecedented strain on public health. Animal models are urgently being developed for SARS-CoV-2 to aid rational design of vaccines and therapeutics. Immunohistochemistry and in situ hybridisation techniques that facilitate reliable and reproducible detection of SARS-CoV and SARS-CoV-2 viral products in formalin-fixed paraffin-embedded (FFPE) specimens would be of great utility. A selection of commercial antibodies generated against SARS-CoV spike protein and nucleoprotein, double stranded RNA, and RNA probe for spike genes were evaluated for the ability to detect FFPE infected cells. We also tested both heat- and enzymatic-mediated virus antigen retrieval methods to determine the optimal virus antigen recovery as well as identifying alternative retrieval methods to enable flexibility of IHC methods. In addition to using native virus infected cells as positive control material, the evaluation of non-infected cells expressing coronavirus (SARS, MERS) spike as a biosecure alternative to assays involving live virus was undertaken. Optimized protocols were successfully applied to experimental animal-derived tissues. The diverse techniques for virus detection and control material generation demonstrated in this study can be applied to investigations of coronavirus pathogenesis and therapeutic research in animal models.

## Introduction

A novel coronavirus, SARS-CoV-2, emerged in Central China late 2019, primarily causing a respiratory disease termed COVID-19^1^. This virus spread rapidly across the globe and was declared a pandemic by the WHO on the 11th of March 2020. As of October 2020, the pandemic has caused 40 million infections including 1 million deaths globally^2^.

SARS-CoV-2 is a *Betacoronavirus* classified under the order *Nidovirales* and family *Coronaviridae*, with a RNA genome that is closely related to SARS-CoV^3^. SARS-CoV-2 and SARS-CoV cellular entry is dependent on the receptor angiotensin-converting enzyme 2 (ACE2). Following the activation of spike protein by the cellular priming proteases TMPRSS2 and cathepsins, fusion of viral envelope and cellular membranes occur, allowing the viral genome to enter the host cell^4^. The spike protein, consisting of S1 and S2 subunits, is a major target for antibody-mediated neutralisation in which immunogenic epitopes reside within the S1 domain^5,6^. During coronavirus replication, nucleoprotein is the most abundantly expressed proteins, followed by the spike^7^. In addition, during the virus replication cycle, double-stranded RNA (dsRNA) is produced as an intermediate product^8^.

The pathogenicity and transmission dynamics of SARS-CoV-2 differs from SARS-CoV^9^ and there is an urgent requirement to address virological and pathological questions using animal models for human disease and for susceptible animal species. Although not an exhaustive list, experimental investigations have been performed in animal models including ferrets^10^, Cynomolgus macaques^11^, Rhesus macaques^12^, African green monkeys^13^, Egyptian fruit bats^14^, golden Syrian hamsters^15^, transgenic hACE2 mice^16^, cats^17,18^ and dogs^18^. Currently, there are limited reports on the use of immunohistochemistry (IHC) and in situ hybridisation (ISH) targeting virus antigens and genes to define virus-host interactions following in vivo experiments^11,14^ due to a scarcity of suitable reagents and tissues for test development.

Here the suitability of antibodies and RNA probes for the detection of SARS coronaviruses present in formalin-fixed paraffin-embedded (FFPE) specimens using IHC and ISH, respectively, is reported. In addition, options for control materials for test development and quality assurance are described. Subsequently, the comparative utility of these optimised assays on tissues from infected animals and the cellular distribution of virus components is demonstrated.

## Results

### Detection of SARS-CoV and SARS-CoV-2 antigens

**Figure 1:**
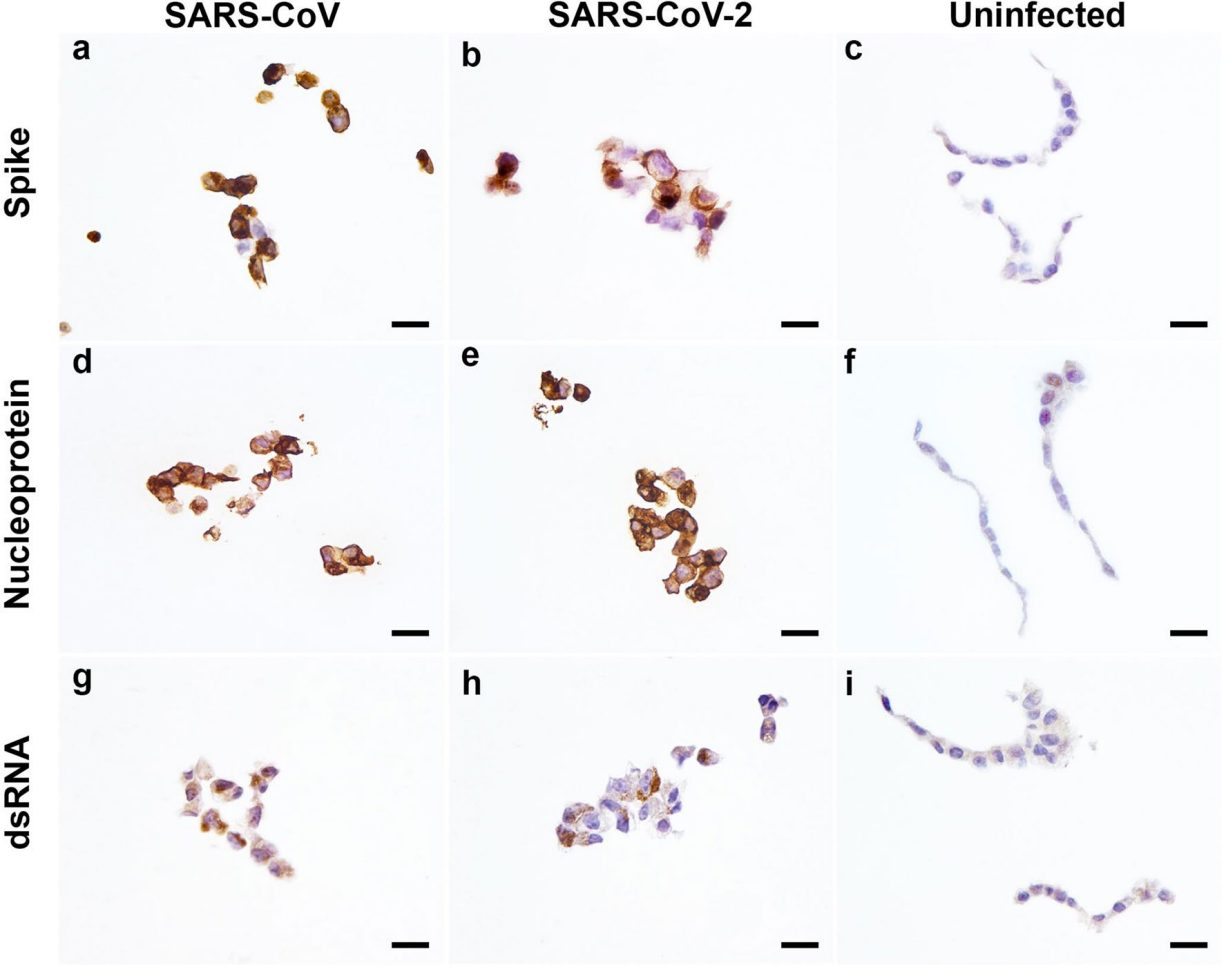
Immunohistochemical labelling of FFPE SARS-CoV and SARS-CoV-2 infected cells and uninfected cells. Immunodetection performed using SARS-CoV spike rabbit monoclonal antibody **(a–c)**, SARS-CoV nucleoprotein rabbit polyclonal antibody **(d–f)** and double-stranded RNA (dsRNA) rabbit monoclonal antibody **(g–i)**. Scale bars, 20 µm.

Several commercially available antibodies to SARS-CoV were evaluated using heat (ph6 or 9) and enzymatic-mediated epitope unmasking technique to detect the presence of virus antigens in FFPE native virus infected cell pellets (Table 1). A rabbit monoclonal (mAb; Sino Biological 40150-R007) spike antibody and rabbit polyclonal nucleoprotein antibody (Sino Biological, 40143-T62) were identified to be suitable for IHC on FFPE cell pellets in which specific cytoplasmic chromogen granules were observed in SARS-CoV and SARS-CoV-2 infected cells but not on uninfected cells (Fig. 1a–f). The rabbit monoclonal (mAb; Sino Biological 40150-R007) spike antibody is compatible with either pH9 or 6 retrieval method, whereas rabbit polyclonal nucleoprotein antibody (Sino Biological, 40,143-T62) can be applied on virus antigen retrieved using either heated pH6 buffer or proteinase (Table 1). The detection of nucleoprotein using the same antibody and antigen retrieval method in a different institution (EMC) also demonstrated reproducible immunolabelling (Supp. Figure 1a, b).

**Table 1:** Antibodies evaluated for immunoreactivity against formalin-fixed paraffin-embedded SARS coronavirus infected cell pellets by immunohistochemistry.

Antibody	Species and clonality	Source	Catalogue number	Immunogen	Suitability with antigen retrievals for detection				Optimal dilution	References
					pH 9	pH 6	Proteinase	No retrieval		
Anti-SARS spike	Rabbit monoclonal	Sino biological	40150-R007	SARS-CoV spike S1 receptor binding domain	Yes	Yes, weaker than pH9	Non-specific	No	1:400–800	Not suitable in earlier report^24^
Anti-SARS nucleoprotein	Rabbit monoclonal	Sino biological	40143-R019	Recombinant SARS-CoV nucleoprotein	Non-specific	Non-specific	Non-specific	n/a	n/a	–
Anti-SARS nucleoprotein	Rabbit polyclonal	Sino biological	40143-T62	Recombinant SARS-CoV nucleoprotein	Non-specific	Yes	Yes	n/a	1:1000–1500	Used in cell pellet and animal tissues^11, 24^
Anti-dsRNA [J2]	Rabbit monoclonal	Absolute antibody	Ab01299-23.0	L-dsRNA	Yes	Yes, weaker than pH9	No	n/a	1:100	–
Anti-dsRNA [J2]	Mouse monoclonal	Scicons	10010500	L-dsRNA	pH 8	No	No	n/a	1:100	^29^
Anti-dsRNA [9D5]	Mouse monoclonal	Absolute antibody	Ab00458-1.1	RDV-RNA-methylated bovine serum albumin complex	No	No	No	n/a	n/a	–
Anti-FIPV	Mouse monoclonal	Invitrogen	FIPV3-70	FIPV	No	No	No	n/a	1:100	^34^

*n/a* not applicable, *dsRNA* double stranded RNA, *FIPV* feline infectious peritonitis virus.

Alongside developing the IHC technique to detect SARS-CoV specific antigens, the IHC detection of dsRNA—a viral replicative intermediate was evaluated. Among the three antibodies evaluated, both the J2 recombinant clone raised in mouse and rabbit was able to detect dsRNA in infected cell pellets with cytoplasmic chromogen deposits (Fig. 1g,h; Supp. Figure 1c, d). However, the amount of immunolabelling was not abundant in comparison to SARS specific antigen detection method. The other clone, 9D5, did not generate chromogen deposits with IHC. The cell pellets were also evaluated for non-specific binding using an alphacoronavirus antibody against Feline infectious peritonitis virus (FIPV). No chromogen was detected in uninfected and SARS-CoV infected cells using the FIPV antibody (not shown).

### Detection of RNA encoding SARS-CoV and SARS-CoV-2 spike protein

**Figure 2:**
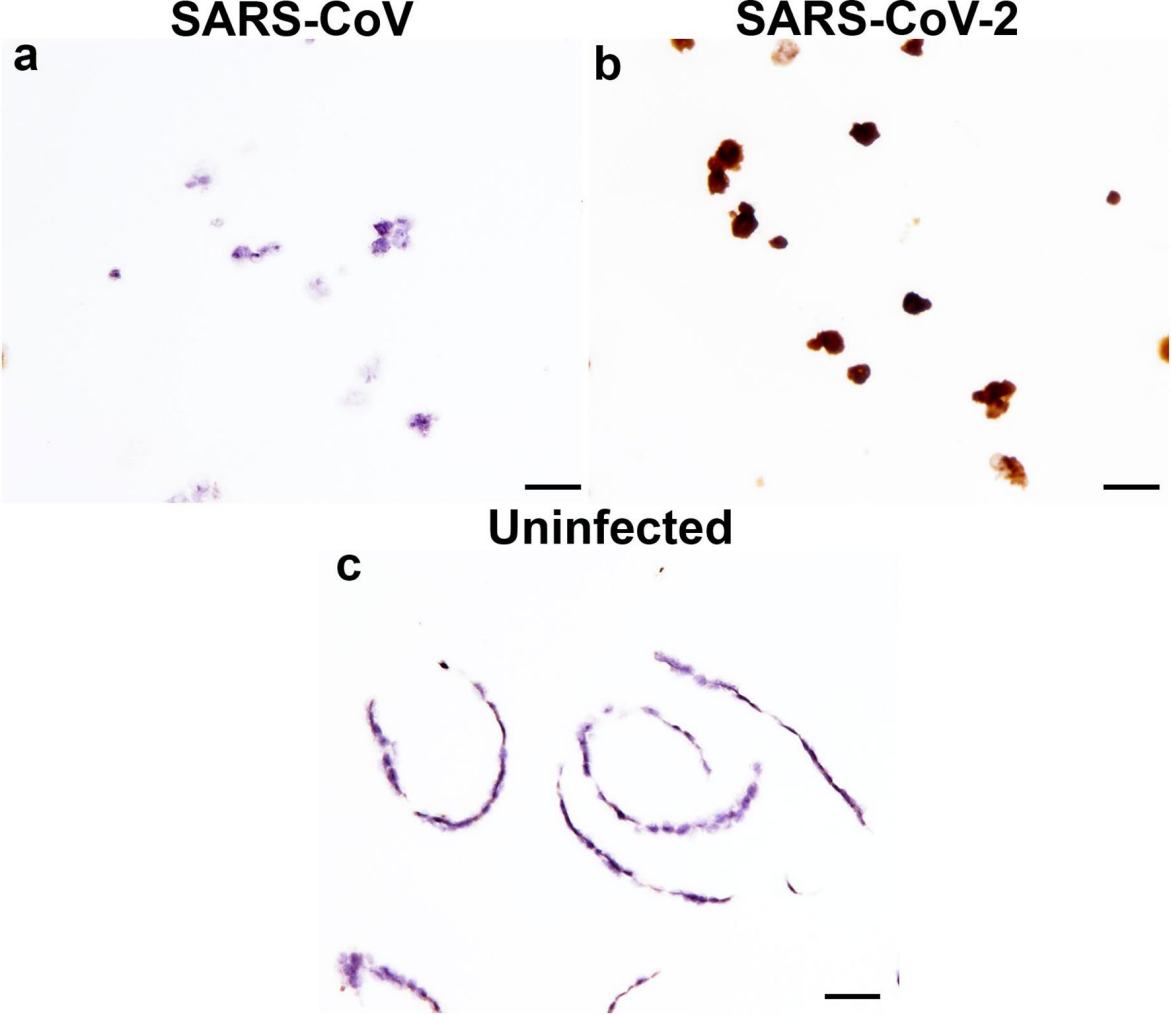
In situ hybridisation (ISH) of FFPE cells infected with SARS-CoV and SARS-CoV-2 using RNAScope^®^. ISH performed using RNA probes designed specific to SARS-CoV-2 spike RNA. SARS-CoV **(a)** and SARS-CoV-2 infected cells **(b)**, uninfected cells **(c)**. Scale bars, 20 µm.

One RNAScope® probe was evaluated for the ability to detect SARS coronavirus RNA in FFPE cell pellets. The V-nCoV2019-5 probe did not produce labelling to SARS-CoV (Fig. 2a) but successfully labelled SARS-CoV-2 infected cell pellets (Fig. 2b). Labelling was not observed on uninfected cell pellets (Fig. 2c).

### Detection of SARS-CoV and SARS-CoV-2 pseudotype virus in producer cells

**Figure 3:**
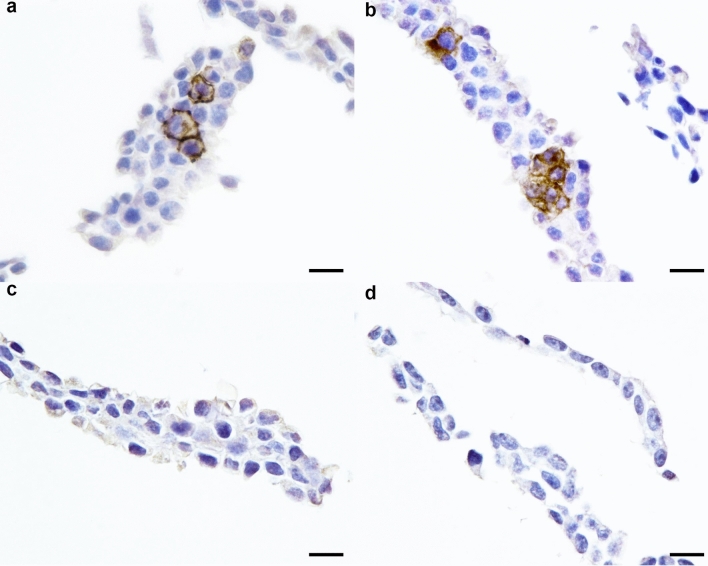
Immunohistochemistry labelling of FFPE cells expressing SARS-CoV, SARS-CoV-2 and MERS spike proteins. Immunodetection performed using SARS-CoV spike rabbit monoclonal antibody on producer cells for SARS-CoV **(a)**, SARS-CoV-2 **(b)** and MERS-CoV pseudotype virus **(c)** and non-transfected cells **(d)**. Scale bars, 20 µm.

To determine if FFPE in vitro generated pseudotype virus expressing recombinant spike protein would be suitable for IHC detection, IHC using the spike mAb identified above was performed on producer cells consisting of lentiviral pseudotype virus expressing either SARS-CoV, SARS-CoV-2 or MERS spike protein. In this assay, the spike mAb was able to detect both SARS-CoV and SARS-CoV-2, displaying specific cytoplasmic and membranous chromogen deposits (Fig. 3a,b). Immunolabelling was not detectable for MERS spike expressing cells (Fig. 3c) or untransfected cells (Fig. 3d).

### Application of IHC and ISH on animal tissues

**Figure 4:**
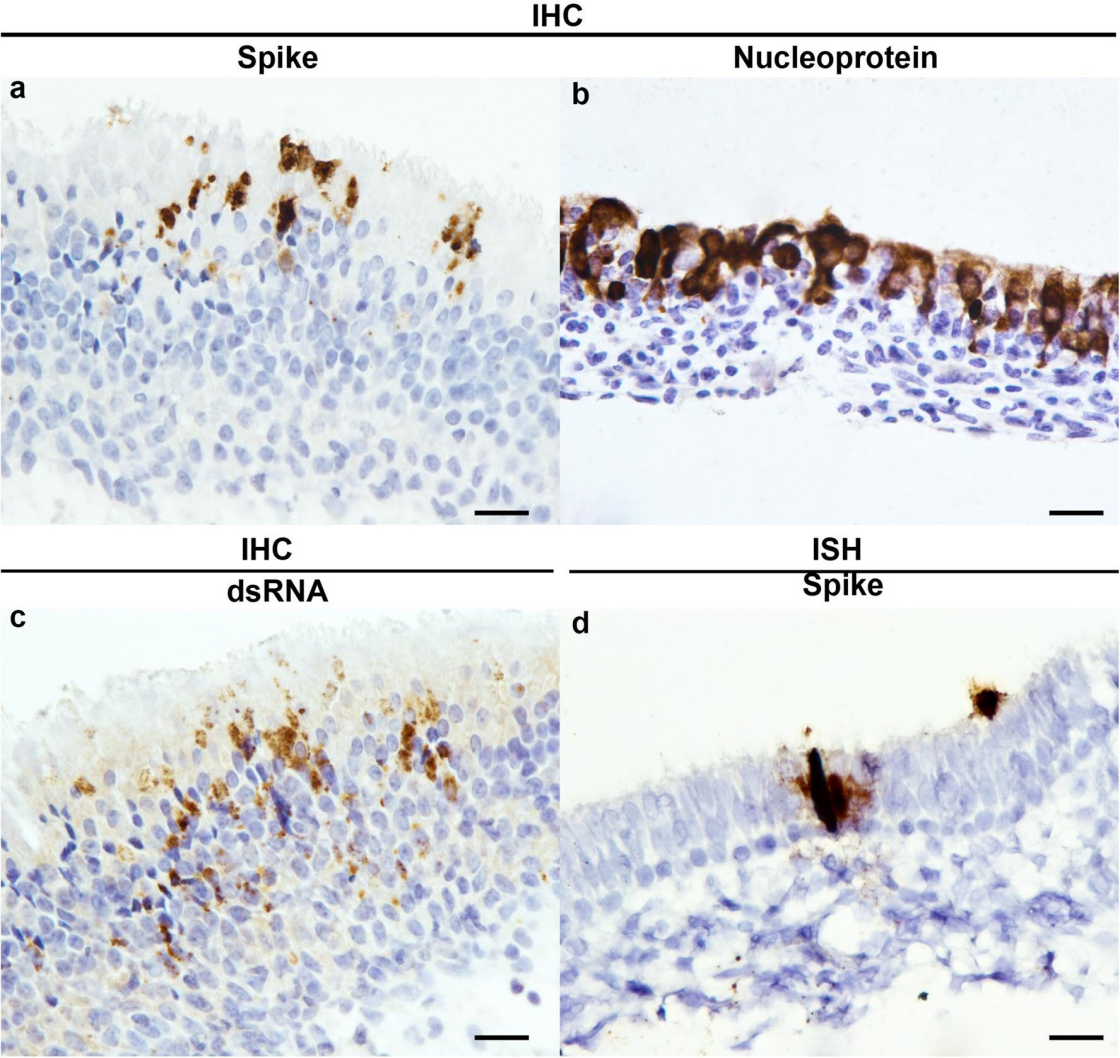
Immunohistochemistry and in situ hybridisation detection of SARS-CoV-2 and RNA on infected ferret tissues. Detection of spike protein **(a)**, nucleoprotein **(b)** and dsRNA antigens **(c)** and spike RNA **(d)** labelling. Tissue shrinkage artefact with ISH pre-treatment **(d)**. Scale bars, 20 µm.

IHC and ISH methods developed and optimised on FFPE cell pellets were tested on nasal turbinates of experimentally derived SARS-CoV-2 infected ferret. Using the spike antibody, immunolabelling was observed specifically labelling the luminal cells in the olfactory epithelial mucosa (Fig. 4a). Nucleoprotein labelling (Fig. 4b) was more ubiquitous in the cytoplasm compared to spike labelling. dsRNA immunolabelling was limited to cytoplasm of the perinuclear region (Fig. 4c), which corresponds to coronavirus replication site^19^. As for ISH against spike gene, chromogen was deposited diffusely within the cytoplasm of the infected epithelial cells (Fig. 4d). Serial sections immunolabelled with nucleoprotein, spike or dsRNA antibody (Fig. 4a,c), or spike ISH showed consistent labelling in infected cell population, confirming the specificity of the detection of SARS-CoV-2 in animal tissues.

## Discussion

In this report, we described optimized methods for antigen and RNA detection for SARS-CoV and SARS-CoV-2 present in FFPE specimens. Using antibodies raised against SARS-CoV spike and nucleoprotein, we were able to detect the antigens of both SARS-CoV and SARS-CoV-2 present in infected cells and processed for histology. In addition, RNAScope^®^ probe designed specifically for SARS-CoV-2 labelled specifically to cognate virus strain. The detection of a virus replication product, dsRNA, was also evident on IHC. Furthermore, we utilised FFPE pseudotype virus producer cells for SARS-CoV spike IHC and demonstrated the specificity of immunolabelling for both strains of SARS virus.

In IHC or ISH applications, positive control materials are needed to be developed and validated for pathology investigations to ensure the quality of results produced. Susceptible cell lines infected with native virus can be generated for such purpose without a requirement for animal tissues. However, this can be problematic for laboratories that lack high containment facilities required to conduct in vitro work. As an alternative, institutions with the relevant genetic engineering expertise can generate proteins expressed in cell culture through transfection and pseudotype system^20^, which can be used as an alternative to live virus work to demonstrate the detection of expressed viral proteins following formalin fixation and paraffin embedding.

Although formalin can reliably inactivate infectious agents, fixation can lead to cross-linking of epitopes and impede antibody binding. Enzymatic- or heat-mediated (pH 6 or 9 buffer) antigen retrieval can be applied to re-expose epitopes although the biochemical impact of these methods have not been assessed^21^. Often, only a single antigen-retrieval method, commonly heat-mediated retrieval, is described for the detection of SARS-CoV-2 virus antigen^22,23^. The spike antibody used in our current study was previously reported as being unsuitable for IHC on FFPE sections^24^. Here, pH 9 retrieval was demonstrated to be more efficient than pH 6 when attempting to unmask spike epitopes. As for nucleoprotein detection, pH 6 antigen retrieval was reproducible in two separate institutional settings. Further, the suitability of protease antigen retrieval for the detection of nucleoprotein was demonstrated in our study. The versatility of heat- or enzymatic-mediated antigen retrieval for nucleoprotein antigen can provide greater flexibility for investigator to perform multilabelling against other antigen of interest.

As there is growing scientific interest in comparing the pathogenesis of SARS-CoV and SARS-CoV-2, we evaluated the ability of antibodies to cross-react to both virus strains. The spike S1 receptor binding domain (RBD) specific antibody was able to detect spike antigen in both native virus and expressed spike protein of SARS-CoV. Although the RBDs of SARS-CoV and SARS-CoV-2 share only 76% amino acid identity^25^, the detection with a single preparation of antibody suggests epitope conservation between the two strains assessed. In contrast, the observation that the SARS-CoV spike S1 antibody did not cross react and bind to the MERS spike protein is likely due to the low amino acid homology (33%) between SARS-CoV-2 and MERS-CoV^26^.

Conversely, the SARS coronavirus nucleoprotein amino acid is highly conserved between SARS-CoV and SARS-CoV-2 with 90% amino acid identity^27^. This fact was supported by our finding that the nucleoprotein antibody cross-reacted with both viruses. Apart from detecting virus-specific proteins, the virus replicative intermediate dsRNA can also be used to detect RNA viruses including coronaviruses^28^. In addition to the ability to detect SARS-CoV as described previously^28^, SARS-CoV-2 can also be detected by IHC using a dsRNA antibody. However, this will require infected cells as positive controls as dsRNA is only synthesised during virus replication.

The utility of these assays was also demonstrated in the detection of viral products in experimentally infected SARS-CoV-2 animal tissues. In the nasal turbinate epithelial cells, dsRNA was present in the peri-nuclear cytoplasm. Early virus replication involves the synthesis of negative-stranded genome template and dsRNA replicative intermediate in which dsRNA was previously observed in the double-membrane vesicles of modified ER membranes situated in the perinuclear region using immungold staining technique^19^. Our study demonstrated spike glycoprotein detection at the apical aspect of respiratory epithelial cells. The localisation was similar to a recent report of SARS-CoV-2 infection of intestinal organoids^29^.

In conclusion, we have demonstrated the suitability of agarose-embedded infected or transfected cell pellets expressing target proteins, fixed and routinely processed for histopathology, in the development of in situ detection methods for the application in animal model research. This preliminary evaluation of virus detection in histological sections has demonstrated suitability of IHC and ISH on FFPE tissues, and importantly has provided knowledge on the spatial cellular distribution of virus components by conventional light microscopy. The detection of SARS coronavirus antigens and nucleic acids in a cell pellet platform also allowed the rapid development of pathology tools that can be applied in future assessments of SARS-CoV-2 pathogenesis and intervention studies.

## Materials and methods

### Cells and viruses

Vero E6 cells were maintained in Dulbecco’s modified Eagle’s medium (DMEM, Gibco) supplemented with 10% foetal calf serum (FCS), HEPES, sodium bicarbonate, penicillin (100 IU/mL) and streptomycin (100 IU/mL). Human embryonic kidney (HEK) 293T-17 cells were grown in DMEM supplemented with FCS, penicillin (100 IU/mL) and streptomycin (100 IU/mL). Cells were grown at 37 °C in a humidified CO_2_ incubator. SARS-CoV (isolate HKU-39849)^29^ and SARS-CoV-2 (isolate BetaCoV/Munich/BavPat1/2020; European Virus Archive Global #026V-03883)^29^ were used for in vitro work whilst SARS-CoV-2 (nCoV/Victoria/1/2020) was used for in vivo experimentation^30^. Both SARS-CoV and SARS-CoV-2 were propagated on VeroE6 (ATCC^®^ CRL 1586TM) cells in Opti-MEM I (1X) + GlutaMAX (Thermofisher), supplemented with penicillin (100 IU/mL) and streptomycin (100 IU/mL) at 37 °C in a humidified CO_2_ incubator. Stocks were produced by infecting VeroE6 cells at a multiplicity of infection (MOI) of 0.01 and incubating the cells for 72 h (hours). The culture supernatant was cleared by centrifugation and stored in aliquots at − 80 °C. Investigations using infectious virus were performed within the biosafety level 3 laboratory at the Erasmus Medical Centre (EMC), Netherlands.

### Generation of virus infected cells

Vero E6 cells were inoculated with SARS-CoV and SARS-CoV-2 at a MOI of 0.3. After 24 h post-infection, cells were fixed with 10% neutral buffer formalin (NBF) along with a separate flask of non-infected cells.

### Generation of virus pseudoparticle in producer cells

Recombinant spike protein was pseudotyped onto a lentiviral core as previously described^31,32^. This was conducted in a BSL2 laboratory in APHA. Briefly, HEK 293T-17 cells were transfected with p8.91 (gag-pol), pCAGGS-s (full length SARS-CoV, SARS-CoV-2, or MERS spike), and pCSFLW. At 5 days post-transfection, cells were fixed with 10% NBF.

### Preparation of cells for histology processing

Fixed monolayer of cells from in vitro infection or transfection were scraped off flasks and centrifuged at 1500* g*. Following removal of supernatant, cell pellets were re-suspended in 2% agarose (Sigma) and allowed to set. Agarose-embedded cell pellets were then processed by routine histology method on a histology processor^33^.

### Animal experimental infection

In vivo experimentations were undertaken within the Advisory Committee on Dangerous Pathogens level 3 (ACDP3) certified animal complex of APHA, UK, in accordance to the Animal (Scientific Procedures) Act 1986. The animal experiment was approved by APHA Animal Welfare and Ethical Review Body (AWERB; HOL-PP3405816/1/001). Ferrets were inoculated intranasally with 1 ml of SARS-CoV-2 of 2 × 10^6^ tissue culture infectious dose 50 (TCID_50_). Animals were humanely euthanised at pre-determined time-points and tissues collected into 10% NBF.

### Immunohistochemistry (IHC)

Four micrometre thick sections were dewaxed and rehydrated through xylene and graded alcohol, respectively, quenched for endogenous peroxidase with 3% hydrogen peroxide in methanol (VWR International) for 15 min (minutes) at room temperature (RT), before epitope unmasking using pH6 (10 mM citric acid buffer, adjusted to pH6 with 1 M sodium hydroxide; Fisherscientific), pH9 target retrieval buffer (Envision FLEX, high pH; Dako) for 10 min at 97 °C, or proteinase XXIV (Sigma-Aldrich) for 15 min at RT. Slides were blocked with normal goat serum for 30 min in RT (1/66 dilution; Vector Laboratories) and assembled into cover plates to facilitate IHC using the Shandon Sequenza system (Shandon). Samples were then incubated with a primary antibody at 4 °C overnight or 1 h at RT, followed by incubation with mouse- or rabbit-specific Envision™ polymer (Dako) for 30 min at RT and visualised using 3,3-diaminobenzidine (DAB) (Sigma Aldrich) for 10 min at RT. Tris-buffered saline with Tween (145 mM NaCl, 5 mM Tris(hydroxymethyl)methylamine, 0.1% w/v Tween®20, adjusted to pH7.6 with 1 M HCl; Fisher Scientific; VWR International) were used for rinsing sections between incubations. Subsequently, sections were counterstained in Mayer’s haematoxylin (Surgipath), dehydrated in ethanol and xylene, and glass coverslips mounted using Dibutyl Phthalate Xylene.

### In situ hybridisation (ISH)

Twenty pairs of double Z proprietary RNA probes targeting the spike gene of SARS-CoV-2, (V-nCoV2019-5, catalogue no. 848569) were synthesised by Advanced Cell Diagnostics (ACD, USA). ISH was performed using the RNAScope^®^ 2.5 HD Brown Detection Kit (ACD) as per manufacturer’s instructions. Tissues were dewaxed and hydrated through xylene and alcohol, respectively, treated with RNAscope^®^ hydrogen peroxide (ACD) for 10 min at RT, and heat-mediated retrieval using Target Retrieval Solution (ACD) for 18 min at 97 °C and Protease Plus (ACD) for 10 min at 40 °C. RNA probes were then added to sections to allow hybridisation for 2 h at 40 °C and followed by 6 rounds of amplification with Hybridise Amp (ACD) at 40 °C, alternating between 30 and 15 min incubation, in the HybEZ™ oven (ACD). Slides were washed with 1× wash buffer (ACD) for 2 min at RT between incubations. Signal was detected using DAB. Sections were counter-stained with Mayer’s haematoxylin (Surgipath), dehydrated in ethanol and xylene, and glass coverslips mounted with Cytoseal (ACD).

## Supplementary Information

Supplementary Figure 1.

## Data Availability

All data generated or analysed during this study are included in this published article (and its Supplementary Information files).
