# Supplementary material for: Development of immunohistochemistry and in situ hybridisation for the detection of SARS-CoV and SARS-CoV-2 in formalin-fixed paraffin-embedded specimens
Source: Sci Rep. 2020 Dec 14;10:21894. doi: 10.1038/s41598-020-78949-0 (PMC7736337; doi:10.1038/s41598-020-78949-0)
Supplement: Supplementary file 1 — Supplementary Figure 1. [file 41598_2020_78949_MOESM1_ESM.pdf]

Development of Immunohistochemistry and *In Situ* Hybridisation for the Detection of SARS-CoV and SARS-CoV-2 in Formalin-Fixed Paraffin-Embedded Specimens

Fabian Z. X. Lean[1]*, Mart M. Lamers[2], Samuel P. Smith[3,7] , Rebecca Shipley[3,6], Debby Schipper [2], Nigel Temperton[4], Bart L. Haagmans[2], Ashley C. Banyard[3], Kevin R. Bewley[5], Miles W. Carroll[5], Sharon M. Brookes[3], Ian Brown[3], Alejandro Nuñez [1]

[1] Pathology Department, Animal and Plant Health Agency (APHA), Woodham Lane, New Haw, Addlestone, KT15 3NB, United Kingdom

[2] Department of Viroscience, Erasmus Medical Centre, Rotterdam, Netherlands

[3] Virology Department, Animal and Plant Health Agency (APHA), Woodham Lane, New Haw, Addlestone, KT15 3NB, United Kingdom

[4] Viral Pseudotype Unit, Medway School of Pharmacy, Universities of Greenwich and Kent at Medway, Chatham ME4 4TB, United Kingdom

[5] National Infection Service, Public Health England, Porton Down, Salisbury, SP4 0JG, United Kingdom

[6] School of Life Sciences, University of Sussex, Falmer, Brighton, BN1 9QG, United Kingdom

[7] Institute for Infection and Immunity, St George's Hospital Medical School, University of London, London, SW17 0RE, United Kingdom.

*Corresponding author: Fabian.lean@apha.gov.uk

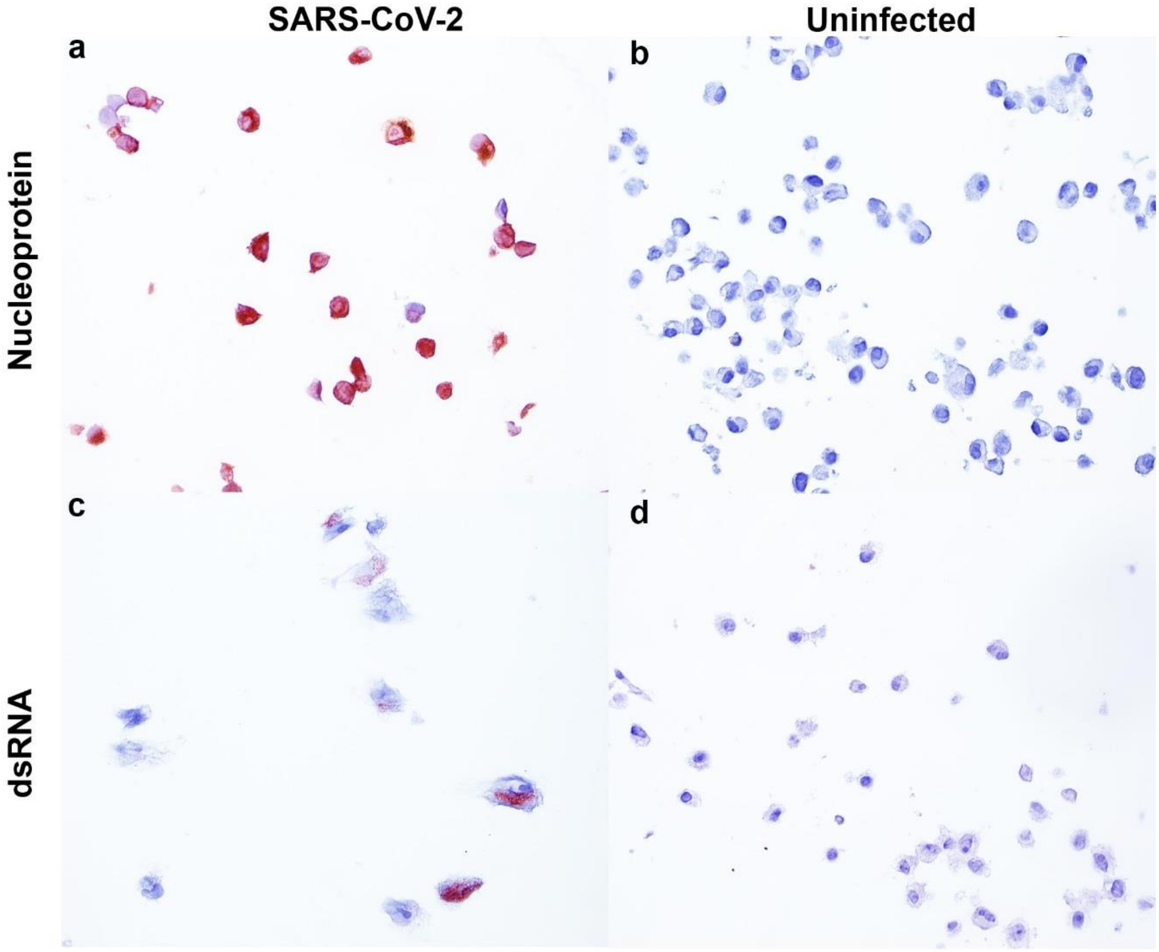

Supplementary Figure 1. Immunohistochemical labelling of FFPE SARS-CoV-2 infected cells and uninfected cells. Immunodetection performed using SARS-CoV nucleoprotein rabbit polyclonal antibody (a, b) and double-stranded RNA (dsRNA) mouse monoclonal antibody (c, d) in Erasmus Medical Centre, Rotterdam. Aminoethyl carbazole (AEC) was used as the chromogen (red). Original magnification 100x.
